# Memory T cells: strategies for optimizing tumor immunotherapy

**DOI:** 10.1007/s13238-020-00707-9

**Published:** 2020-03-27

**Authors:** Qingjun Liu, Zhongjie Sun, Ligong Chen

**Affiliations:** 1 School of Pharmaceutical Sciences, Key Laboratory of Bioorganic Phosphorus Chemistry and Chemical Biology (Ministry of Education), Tsinghua University, Beijing, 100084 China; 2 Newish Technology (Beijing) Co., Ltd., Xihuan South Road 18, Economic & Technical Development Zone, Beijing, 100176 China; 3 Moon (Guangzhou) Biotech Co., Ltd., Room 301, Building B5, Enterprise Accelerator, No. 11 Kaiyuan Avenue, Huangpu District, Guangzhou, 510000 China; 4 Advanced Innovation Center for Human Brain Protection, Beijing Tiantan Hospital, Capital Medical University, Beijing, 100088 China

**Keywords:** memory T cells, tumor immunology, metabolism, gut microbiota

## Abstract

Several studies have demonstrated that memory T cells including stem cell memory (Tscm) T cells and central memory (Tcm) T cells show superior persistence and antitumor immunity compared with effector memory T (Tem) cells and effector T (Teff) cells. Furthermore, the Tcm/Teff ratio has been reported to be a predictive biomarker of immune responses against some tumors. Thus, a system-level understanding of the mechanisms underlying the differentiation of effector and memory T cells is of increasing importance for developing immunological strategies against various tumors. This review focuses on recent advances in efficacy against tumors, the origin, formation mechanisms of memory T cells, and the role of the gut microbiota in memory T cell formation. Furthermore, we summarize strategies to generate memory T cells *in* (*ex*) *vivo* that, might be applicable in clinical practice.

## Introduction

Adoptive cell-transfer (ACT) therapy, which involves the *ex vivo* expansion and reinfusion of tumor-reactive T cells such as tumor-infiltrating lymphocytes (TILs) and, genetically-retargeted T cells with conventional T-cell receptors (TCRs) or chimeric antigen receptors (CARs), is emerging as a potential curative treatment for patients with advanced-stage cancer. A melanoma patient who received *ex vivo* expanded TILs achieved a durable complete response (Rosenberg et al., 2011). However, the dysregulated metabolic activity of tumor cells results in an immunosuppressive tumor microenvironment (TME) including soluble factors secreted by tumor or stromal cells, and inhibitory immune cells such as myeloid-derived suppressor cells (MDSCs) and regulatory T cells (Tregs), which resulted in TILs experiencing metabolic stress and exhaustion (Li et al., 2019). Furthermore, tumor cells limit antitumor responses via immune checkpoints such as CTLA-4 and PD-1 moleculars. To ameliorate negative regulation mediated by immune checkpoint molecular expression by tumor cells, immune checkpoint inhibitor (ICI) antibodies blocking the CTLA-4 or PD-1 pathway have been developed. Most patients who have tumor responses maintain long-lasting disease control, but one-third of patients experience relapse (Ribas and Wolchok, 2018). ‘T cell exhaustion’ has been used relatively recently to describe the response of T cells to tumors and is defined by poor effector function, sustained expression of inhibitory receptors and decreased cytokine levels. It is a state of T cell dysfunction that arises during cancer, resulting from negative immunoregulatory pathways (Blank et al., 2019). However, T cell exhaustion is not irreversible and T cell can be normalized to control tumors through better manipulation (Sanmamed and Chen, 2018). Recently, many studies have found that T cells with memory phenotypes show relatively superior *in vitro* and *in vivo* antitumor function. It has been found that the capacity of T cells to kill tumors is determined by the anti-cancer “base” for stem cell-like CD8^+^ T cell formation, and is not due to many exhausted T cells with positive checkpoints or high expression levels of PD-L1 in the tumor (Jansen et al., 2019). Furthermore, the gut microbiota has emerged as a factor that enhances the anti-tumor efficacy of blocking ICI antibodies by promoting memory T cell formation. Therefore, we speculate that manipulation of tumor-reactive T cells to promote differentiation into memory phenotypes *in vitro* or *in* (*ex*) *vivo*, combined with blocking ICI antibodies, could represent an effective antitumor strategy. This review will summarize the biology of memory T cells, discuss the origin and formation of memory T cells and the immunological mechanisms of their antitumor actions, explore the roles of the gut microbiota in memory T cell formation and cover manipulation techniques to produce T cells with memory phenotypes.

## Phenotypes of memory T cells

The differentiation and activation of T cells is dependent on signals transduced by three different receptors: TCRs (including the CD4 and CD8 receptors that respond to MHC-II displayed antigens and MHC-I displayed antigens, respectively) (Liu and Gao, 2008), costimulatory receptors, and cytokine receptors. These signals drive naïve T cells to differentiate into effector or memory T cells. Upon activation, T cells seek to destroy the source of the cognate antigen, such as infected cells, or tumor cells, by releasing cytokines and directly killing target cells (Smith-Garvin et al., 2009). Naïve T cells are actively maintained in a state of hyporesponsiveness, which is characterized by a small cell size, a low proliferative rate, and low basal metabolism. Naïve T cells are defined by the expression of CD45RA and the lymph node homing markers CD62L and CCR7 (Table 1). These markers enable naïve T cells to extravasate from high endothelial venules and migrate into the T-cell zones of peripheral lymph nodes where they encounter antigens presented by dendritic cells (DCs). Antigen-experienced T cells downregulate CD45RA expression and express CD45RO. After naïve T cells differentiate into Teff cells, they readily release cytotoxic granules and effector cytokines upon engagement of their TCR with the cognate antigen. Teff cells are negative for CD27, CD28, and lymph node homing markers, but express markers of terminal T cell activation such as Killer cell lectin-like receptor subfamily G, member 1 (KLGR-1) and the NK marker CD57 (Sallusto et al., 1999; Klebanoff et al., 2006). In general, the memory T cells include stem cell memory T (Tscm) cells and central memory T (Tcm) cells and effector memory T (Tem) cells, which have different specific phenotypes (Table 1). Tscm cells express increased levels of CD95, IL-2Rβ, CXCR3, and LFA-1 compared with naïve T cell (CD45RA^+^CD45RO^−^CCR7^+^CD62L^+^CD27^+^CD28^+^IL-7Rα^+^) (Gattinoni et al., 2011). Tcm cells and Tem cells are often distinguished by CCR7 expression (Sallusto et al., 1999) and function (Sallusto et al., 2004). Tcm cells (characterized by the CD45RO^+^CCR7^+^CD27^+^CD28^+^CD62L^hi+^ phenotype) generally reside in lymphoid organs and do not have an immediate lytic function, whereas Tem cells are found in nonlymphoid tissues, have lytic activity and are CD62L^lo^CCR7^−^ (Unsoeld and Pircher, 2005). Tem cells express higher levels of receptors responsible for migration to inflamed tissues and have a stronger immediate effector function than Tcm cells (Sallusto et al., 2004) (Geginat et al., 2001). Jeffrey and Igor summarized the markers of the subset of memory CD8^+^ T cells with the highest proliferative capacity upon recall as follows (Ahlers and Belyakov, 2010): CD62L^high^, CD127 (IL-7R)^high^, KLRG1^low^, CD27^high^, CD43^low^, CD122 (IL-2/IL-15Rβ)^high^, and Bcl-2^high^. Recently, CD69^+^CD103^+^ tissue-resident memory T cells (T_RM_), were identified as nonrecirculating non-recirculating immune cells that reside in peripheral tissues, and have been reported to mediate tumor protection by promoting the tumor-immune equilibrium through the secretion of cytokines and/or CD103-enhanced tumor cell killing. Simone et al. systematically discussed the role of T_RM_ cells in cancer immunosurveillance (Park et al., 2019). So we will not repeat that information here.

**Table 1:** Phenotypic markers of memory T cells

	Naïve	Tscm	Tcm	Tem	Teff
CD45RA	+++	++	++	+	−
CD45RO	−	+	+	+	−
CD44	−	+/−	+++	+/−	−
CCR7	+++		+++	+/−	−
CD62L	+++	+++	+++	+/−	−
CD127	+/+++	+++	+++	+	+/−
CD122	+	+++	+++	+	+/−
CD28	++	+++	+++	+	−
CD27	+/−		++	++	+/−
CD43	−	−	−	+/−	+++
CD95	+/−		+/−	++	+++
KLRG1	−	−	−	++	+++
Perforin	−	−	−	++	+++
GranzymeB	−	−	−	+	+++

## Efficacy of memory T cells against tumors

ACT of T cells with a naïve, Tscm or Tcm immunophenotypes has shown superior *in vivo* efficacy in preclinical testing. Compared with known memory cell populations, Tscm cells display an increased proliferative capacity, more efficient reconstitution in immunodeficient hosts, and superior antitumor responses in a humanized mouse model (Gattinoni et al., 2011). Tcm cells produce higher levels of cytokines, have stronger cytotoxic activity *in vitro*, and show a superior ability to eradicate established tumors in mice compared with Teff cells (Sallusto et al., 1999; Klebanoff et al., 2006). Similar results have been obtained in a nonhuman primate model: Tcm cells persisted longer than Tem cells *in vivo* (Berger et al., 2008). Furthermore, T cells with a naïve phenotype from TCR transgenic mice demonstrated enhanced antitumor activity following ACT compared with their mature T cell counterparts (Hinrichs et al., 2009). Mouse CD8^+^ T cells cultured in the presence of IL-2 and IL-15 (the latter promotes T cell differentiation into memory phenotypes) show greater tumor cytotoxicity than cells cultured in the presence of IL-2 alone (Klebanoff et al., 2004; Klebanoff et al., 2005). Tcm (CD3^+^CD62L^+^CD45RO^+^) cells cultured *ex vivo* by the Newish company after isolation from peripheral blood mononuclear cells (PBMC) of hepatocellular carcinoma (HCC) patients could effectively kill the human HCC cell line, QGY-7703, while relatively high levels of IFN-γ and TNF-α were secreted in the process of Tcm activation. Furthermore, the Tcm also significantly inhibited the subcutaneous growth of QGY-7703 and SMMC-7721 tumors in nude mice (unpublished data). Professor Hong Zhao conducted a clinical trial (Clinicaltrials.gov ID: NCT03575806) using Tcm to control HCC with microvascular invasion (MVI) after radical resection at the Cancer Institute and Hospital, Chinese Academy of Medical Sciences. Midterm clinical trial results demonstrated that Tcm significantly extended the median relapse free survival (RFS) of HCC patients (21.7 months vs. 18.43 months, *P* = 0.049) (unpublished data).

The superior antitumor efficacy of memory T cells may be associated with several factors. First, memory T cells have lower activation thresholds than naïve T cells: these cells can respond to 100-fold lower doses of antigen and respond more rapidly in the presence of costimulation. Depending on the antigen dose and level of costimulation, naïve T cells require 6 h to ≥30 h of TCR stimulation to achieve activation, while memory T cells become activated within 0.5 h to 2 h (Lanzavecchia and Sallusto, 2001). However, CD8^+^ Tcm cells exhibited increased antigen threshold requirements for recall responses, resulting from both decreased surface TCR expression and increased protein tyrosine phosphatases expression compared with naïve T cells (Mehlhop-Williams and Bevan, 2014). Second, memory T cells have an enhanced capacity to migrate to lymph nodes. Because they express the lymph node homing receptor CCR7, memory T cells can migrate to lymph nodes where DCs present antigens to them in the context of MHC molecules. Third, memory T cells persist for very long durations. Some studies have reported that Tcm cells can survive for longer than 10 years. Fourth, memory T cells mediate primary immunosurveillance of peripheral tissues. Tcm cells are present in healthy, noninflamed human skin, lung, colon, and cervix tissues, and have potent effector functions. Finally, the Tregs present in the TME have a relatively weak ability to inhibit the functions of memory T cells (Yang et al., 2007).

Several studies have demonstrated that the rapid recall ability of memory T cells is mediated by the ability of transcription factors to bind to DNA encoding appropriate genes, which is in turn regulated epigenetically by the local chromatin state. NF-κB is activated and translocates to the nucleus at similar levels in both naïve and memory T cells but is only able to bind DNA and induce the expression of *Ifng* in memory T cells (Lai et al., 2011). It has been demonstrated that the abilities of naïve, Tcm and Tem of human CD4^+^ subsets to produce cytokines are associated with the presence of positive chromatin modifications at promoters and enhancers of these genes (Barski et al., 2017).

The high numbers of CD8^+^ T cells and CD45RO^+^ memory T cells within a primary tumor lesion remarkably correlate with positive clinical outcome in different cancers (Fridman et al., 2012). As previously mentioned, the increased frequencies of Tcm cells (CD4^+^ and CD8^+^) and enhanced tumor inflammation profiles in melanoma and non-small cell lung cancer (NSCLC) (Manjarrez-Orduno et al., 2018) are congruent with reports that Tcm cells are the primary repositories of the immunogenic experiences of a lifetime (Wherry et al., 2003; Berger et al., 2008). Thus, the Tcm/Teff ratio was proposed as a predictive biomarker of response to checkpoint inhibitors in NSCLC patients (Reboursiere et al., 2018).

## Origin of memory T cells

**Figure 1:**
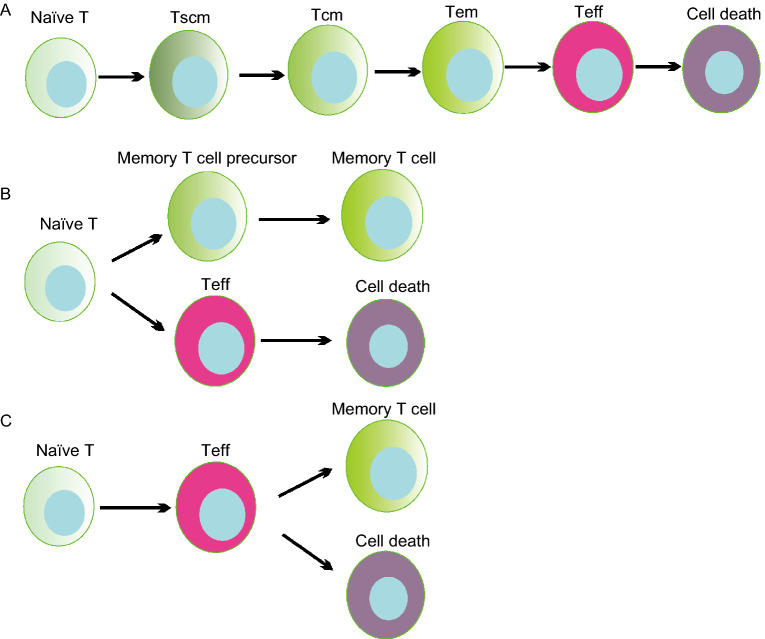
**Three models of memory T cell formation**. (A) Memory T cells differentiate from naïve T cells in a stepwise manner as they progress from Tscmto Tcm, and then Tem, a more terminally differentiated phenotype. (B) Memory T cells are from discrete subsets that contain memory precursor genes in the naïve T cell population. (C) Memory T cells are derived from surviving effector T cells as the methylation patterns in memory T cells are similar to those in effector T cells

Three models have been proposed to describe memory T cell formation (Fig. 1). In the first model, Tscm, Tcm and Tem cells differentiate from naïve T cells in a stepwise manner as they progress toward a more terminally differentiated phenotype (Sallusto et al., 1999) (Fig. 1A). A study in support of this model identified a subset of memory T cells with KLRG1^int^ expression that were unique in their ability to produce IL-2 (Sarkar et al., 2008). In the second model, memory T cells are predetermined by numeric differences in TCR clonotypic precursor frequencies and intraclonal diversification (Fig. 1B). In the third model, memory T cells arise during the contraction phase of the immune response and develop directly from effector cells (Fig. 1C). Two papers published in Nature provided strong evidence that memory T cells are generated from effector T cells through epigenetic modifications, and revealed that Dnmt3a works as a key DNA methyltransferase in driving memory T cell formation (Akondy et al., 2017; Youngblood et al., 2017). Several studies have shown that the self-renewal properties of antigen-specific memory T cells are maintained through asymmetrical mitoses similar to those of pluripotent stem cells (Chang et al., 2007; Haining et al., 2008; Tomayko et al., 2008). Overall, the third model might be the most widely accepted.

## Mechanisms of memory T cell formation

**Figure 2:**
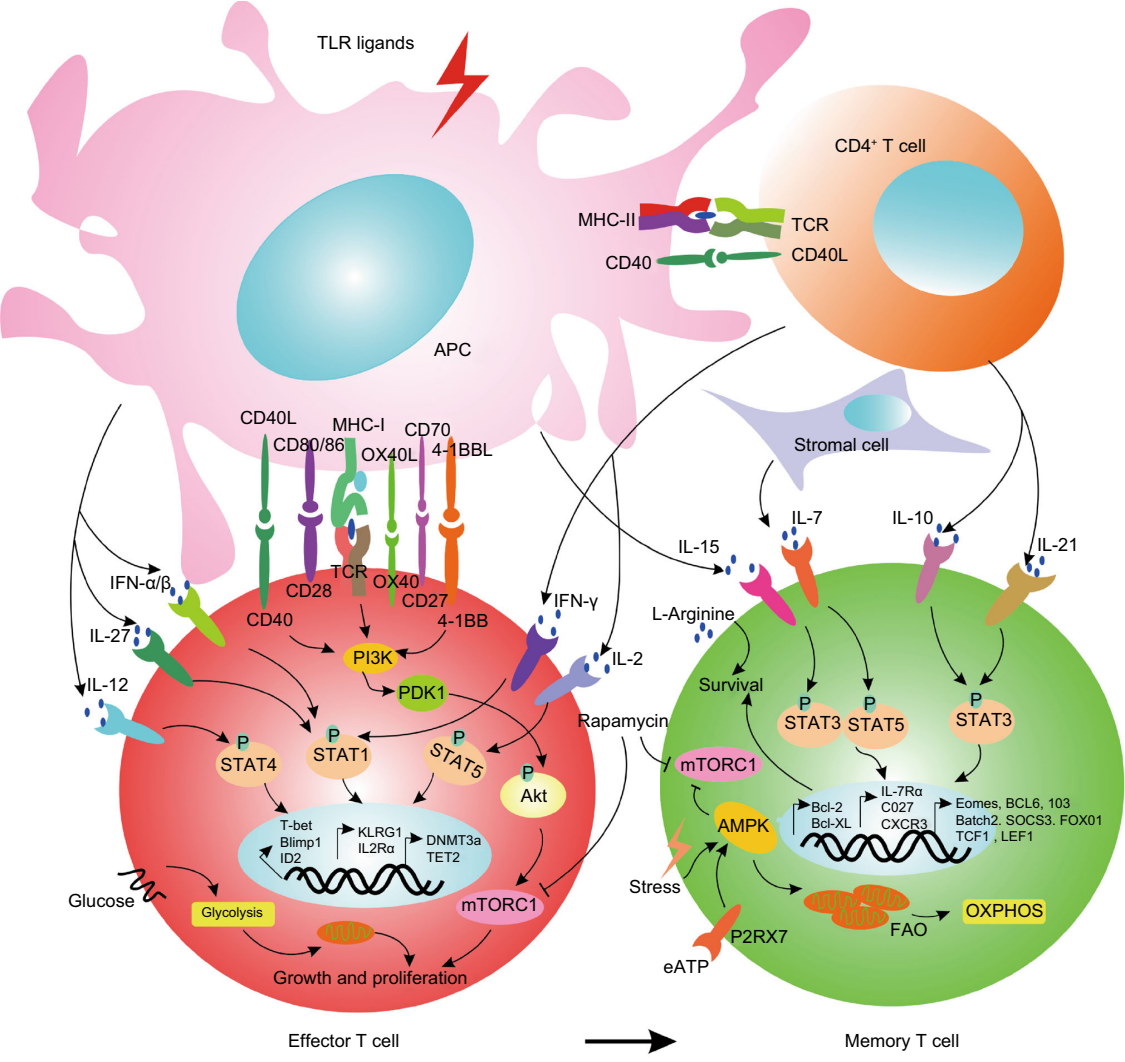
**Molecular and metabolic regulation of effector and memory T cell differentiation**. Following immunization or infection, naïve CD4^+^ T cells are activated by cognate antigens presented by DCs through MHC class II and upregulated CD40L expressions. Then, DCs are licensed by cognate CD4^+^ T cells through a CD40-CD40L interaction, which enables the DCs to obtain sufficient antigen-presenting and co-stimulation capacities to induce a robust CD8^+^ T cell response. In some cases, DC functional maturation is mediated by TLR ligands and bypasses the requirement for CD4^+^ T cells. The differentiation of effector and memory T cells is orchestrated by three major signals: TCR, co-stimulatory molecules and cytokines. Effector T cells formation initiates with TCR signals delivered by DCs through MHC class I in the presence of antigen, while memory T cells are generated after the antigen is quickly cleared. Integration of TCR signals, co-stimulatory signals including CD28-CD80/CD86, CD40-CD40L, OX40-OX40L, CD27-CD70 and 4-1BB-4-1BBL plays important roles in certain inflammatory settings. Cytokines can affect T-cell differentiation, proliferation and survival at many stages of the immune response. For example, IFN-γ α/β, IL-27, and IL-12 derived from mature DCs and IFN-γ and IL-2 derived from helper CD4^+^ T cells activate the JAK-STAT signaling pathway mediated through STAT1/STAT4 or STAT5 in effector T cells, while IL-10 and IL-21 secreted by helper CD4^+^ T cells selectively activate STAT3 and STAT5 in memory T cells. In addition, memory T cells are maintained in an antigen-independent, cytokine-independent manner mainly through the action of stromal cell derived IL-7 and DC derived IL-15, which promotes cell survival by upregulating the levels of anti-apoptotic proteins such as BCL-2 and BCL-xL. These three classes of signals are closely linked and act collaboratively to endow T cells with different transcriptional profiles. Therefore, effector T cells, characterized as KLRG1^high^ IL7Rα^low^ IL2Rα^high^ express high level of transcriptional factors such as T-bet, Blimp1, and ID2, and epigenetic regulators including DNMT3a and TET2. In contrast, memory T cells characterized as KLRG1^low^ IL7Rα^high^ CD27^high^ CXCR3^high^ express high level of EOMES, BCL-6, ID3, BATCH2, SOCS3, FOXO1, TCF1 and LEF1. Additionally, effector and memory T cell differentiation are coupled with metabolic reprogramming. In effector T cells, activation of the PI3K-AKT-mTOR pathway promotes aerobic glycolysis, a hall-mark of activated T cells. In contrast, in memory T cells, cellular stress, such as growth factor deprivation or a low ratio of ATP/AMP, will activate AMPK and inhibit mTOR signaling. Moreover, extracellular ATP released from dying cells can activate P2RX7, which further induces AMPK expression and mitochondrial homeostasis. As a result, anabolism is shut down, and metabolism switched to fatty acid oxidation (FAO) and oxidative phosphorylation (OXPHOS). Accordingly, pharmacological inhibitors of mTOR pathway such as rapamycin can be useful to memory T cell induction. Recently elevating L-arginine level was also reported to promote the metabolic shift from glycolysis to oxidative phosphorylation and promote cell survival, providing another potential strategy for memory T cell generation *in vitro*

The transition from antigen-activated CD8^+^ T cells to memory T cells depends upon multiple sequential signals, including the strength and duration of TCR stimulation (Williams et al., 2008), CD4^+^ T cell help (Kaech et al., 2002; Hikono et al., 2007; Intlekofer et al., 2007; Joshi et al., 2007; Pearce and Shen, 2007; Schulz et al., 2009), costimulation (the CD28 family (Fos et al., 2008) including CD28, CTLA-4 (Kocak et al., 2006), ICOS, TIM-3, BTLA (Krieg et al., 2007) and PD-1/2; the TNF superfamily including CD40 (Ahlers et al., 2002; Ahonen et al., 2004; Huster et al., 2004; Sanchez et al., 2007), CD27 (Hendriks et al., 2005; Dolfi et al., 2008), CD30, 4-1BB (Hendriks et al., 2005; Kocak et al., 2006; Sabbagh et al., 2008), OX40 (Hendriks et al., 2005) and TNF2), and the presence of cytokines (IFN-γ, IL-21, IL-7 and IL-15) (Pearce and Shen, 2007; Schulz et al., 2009) that regulate survival (Fig. 2). TCR recognition of the peptide: MHC complex results in rapid downregulation of CD62L, SIP1, and IL-7R expression, upregulation of activation markers expression, and acquisition of effector functions. Although signaling induced by MHC-I ligands is not required for CD8^+^ memory T cell survival (Murali-Krishna et al., 1999; Jabbari and Harty, 2005), it is still conceivable that TCR interaction with low-avidity self-ligands, or ligand-independent signaling, could be involved in memory (Polic et al., 2001). Recently, Jansen et al. demonstrated that stem-like T cells resided in dense antigen-presenting cell niches within tumors, and that tumors that failed to form these structures were not extensively infiltrated by T cells (Jansen et al., 2019). It has been confirmed that DCs play a very important role in memory T cell formation in tumors. CD4^+^ T cells help enlarge the initial CD8^+^ T cell response and program the differentiation of responding CD8^+^ T cells to generate long-lived, protective memory (Bevan, 2004). What is more surprising is the evidence that CD4^+^ T cells are also required long after antigen elimination to maintain CD8^+^ T cells (Sun et al., 2004; Badovinac et al., 2006; Williams and Bevan, 2007). Endogenously activated CD4^+^ T cells that express low levels of CD25 and produce numerous cytokines are key to maintaining memory CD8^+^ T cells (Setoguchi et al., 2005). In mouse CD4^+^ T cells, initial TCR signaling works similarly in naïve and memory cells. However, a key kinase, ZAP-70, is phosphorylated to a lesser degree in memory T cells than in naïve T cells (Farber et al., 1997), suggesting that memory T cells actually receive weaker signaling through their TCRs. Nevertheless, a study reported that ZAP-70 phosphorylation was not different between in naïve and memory CD8^+^ T cells. However, an increased LAT concentration and phosphorylation in memory T cells led to increased ERK and Jun phosphorylation following activation (Kersh et al., 2003). Another study demonstrated that SLP-76 was phosphorylated to a lesser degree in memory T cells than in naïve cells, again suggesting weaker TCR signaling in memory T cells (Hussain et al., 2002).

IL-7 and IL-15 play very important roles in memory T cell formation by regulating survival. The IL-15 receptor is a three-chain complex including IL-15Rα and two signaling components shared with IL-2R: IL-2Rβ (CD122) and the common γ chain (CD132). The IL-15Rα chain must be expressed in the cell that produces IL-15, suggesting that IL-15α functions as an intracellular transporter for the cytokine and presents IL-15 in a membrane-bound form to recipient cells (Klonowski and Lefrancois, 2005; Surh and Sprent, 2005; Ma et al., 2006). Recent studies have suggested that the binding of IL-15 to IL-15α results in a complex that greatly potentiates signaling via the two-chain IL-2/15Rβγ receptor (Mortier et al., 2004; Mortier et al., 2006; Quemener et al., 2006; Rubinstein et al., 2006). Soluble IL-15 upregulates the expression of 4-1BB (CD137) on memory CD8^+^ T cells (Pulle et al., 2006). Type I IFNAR/STAT1 signals can upregulate IL-15 production by DCs and have been shown to fuel a feed-forward loop required for CD8^+^ T-cell survival during the contraction phase and for T-cell memory cell formation (Kolumam et al., 2005; Whitmire et al., 2007; Bell et al., 2008). The transcription factors Foxo1 and KLF2, which are regulated by posttranscriptional modifications, coordinate renewed expression of IL-7R, CD62L, CCR7, and S1P1, and downregulate inflammatory chemokine receptors expression, on cells destined to become Tcm cells. Limited growth factors levels cause activated T cells to shut down growth and proliferative programs sustained through the TCR/CD28, IL-2 and P13K/AKT/mTOR pathways and to upregulate autophagy pathways during trafficking into nonactivated lymphoid tissue and tissue niches. In these sites, Tcm and Tem cell populations become dependent upon cytokines and tissue-specific interactions for maintenance and homeostasis. CD8^+^ T cells that receive CD4^+^ T cell help in the T cell rich zones of draining lymph nodes may be destined for long-term survival. It is very interesting that the bone marrow (BM) represents an elective site for the maintenance of antitumor memory T cells. A study showed that athymic nu/nu mice inoculated with syngeneic dormant tumor cells isolated from the BM (quiescent tumor cells) maintained the persistence of a high frequency of specific CD8^+^ T cells in the BM and spleen. Furthermore, CD44^hi^ memory T cells from the BM showed a significantly higher turnover rate than corresponding cells from the spleen or lymph nodes (Mahnke et al., 2005). A similar result was found in breast cancer patients. The breast cancer patients showed higher proportions of memory CD4^+^ and CD8^+^ T cells in the BM than healthy subjects. Patients with disseminated tumor cells in their BM had more memory CD4^+^ T cells and more CD56^+^CD8^+^ cells than patients with tumor cell-negative BM (Feuerer et al., 2001). Both studies demonstrated that the microenvironment of the BM containing a low level of antigen favors the maintenance of Ag-specific memory T cells. The balance between costimulatory signals that upregulate antiapoptotic factors expression, and negative costimulatory molecules upregulated during the effector phase that block effector function, such as CTLA-4, BTLA-4, and PD1 (during chronic stimulation) ultimately determines the population of cells that survives and becomes the memory pool (Fig. 2 and Fig. 2 of the reference (Ahlers and Belyakov, 2010)).

Metabolic activity is intimately linked with T cell fate and function (Fig. 2). Elevated L-arginine levels induce global metabolic changes, including a shift from glycolysis to oxidative phosphorylation, in activated T cells. Elevated L-arginine levels also promote the generation of Tcm cells endowed with a relatively high survival capacity and, in a mouse model, relatively high antitumor activity. Three transcriptional regulators (BAZ1B, PSIP1, and TSN) sense L-arginine levels and promoted T cell survival (Geiger et al., 2016). P2RX7 promotes mitochondrial homeostasis and metabolic function in differentiating memory CD8^+^ T cells, at least in part by inducing AMP-activated protein kinase expression (Borges da Silva et al., 2018). Thus, P2RX7 is required for the establishment, maintenance and functionality of long-lived central and tissue-resident memory CD8^+^ T cell populations. Bcl-6, a transcriptional repressor and antiapoptotic factor, is necessary for the generation of antigen-specific CD8 T-cell memory and may function by suppressing Blimp-1 mediated activation-induced cell death (AICD) (Ichii et al., 2004). IL-15 can also prevent AICD.

The role of tumor specific CD8^+^ T cells in tumor rejection is well established, thus the fact that many studies focus on the mechanisms underlying memory CD8^+^ T cell formation is comprehensible. However, the role of CD4^+^ T cells in the antitumor response in addition to supporting memory CD8^+^ T cell formation is also very important. It has recently been reported that spontaneous and immunotherapy-induced antitumor responses require the activity of tumor antigen-specific CD8^+^ and CD4^+^ T cells, even in tumors that do not express MHC-II molecules, indicating that CD4^+^ T cells, which recognize antigens displayed by MHC-II, are necessary to eliminate tumors (Alspach et al., 2019). Furthermore, we found that IL-15 could induce human CD4^+^ T cells to develop memory phenotypes more easily than human CD8^+^ T cells, the induced memory CD4^+^ T cells could respond to DCs pulsed with tumor antigens (Data not shown). Recently, it has been found that the gut microbiota can promote memory CD4^+^ T cell formation in tumor-bearing mice and cancer patients (Pitt et al., 2016). We believe that an increasing number of studies will focus on the mechanisms underlying memory CD4^+^ T cell formation in the future.

Overall, the molecular mechanisms underlying memory T cell formation are very complicated, and we clearly depict them in the review as much as possible (Fig. 2). With the deepening and development of research, we believe that the molecular mechanisms underlying memory T cell formation will be made clearer.

**Figure 3:**
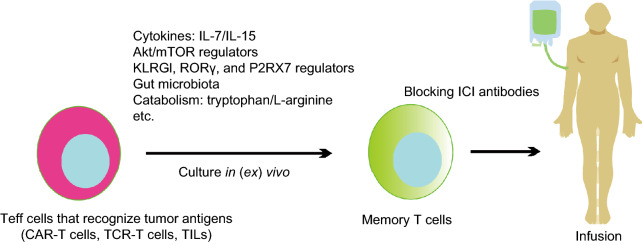
**Methods to transform Teff cells into T cells with a memory phenotype by conditional culture*in*(*ex*)*vivo***. These approaches would endow T cells specific to tumor antigens with superior persistence and antitumor immune function in cancer patients

## The role of the gut microbiota in memory T cell formation

The gut microbiota contributes to digestion, epithelial barrier integrity, and mucosal immunity. Though the ‘mucosal firewall’ confines microbial antigens to the gut-associated lymphoid tissue, dendritic cells drive T cell differentiation in response to dietary antigens and commensal bacteria. Vast numbers of potentially commensal-reactive effector and memory T cells populate the intestinal mucosae in the context of unique human exposure to pathogenic and nonpathogenic microorganisms (Farber et al., 2014). It has been suggested that the role of immune memory in vertebrates is to preserve proper immune homeostasis with commensal microorganisms through coevolution (McFall-Ngai, 2007). Studies in mouse models have shown that the presence and composition of the microbiome are crucial in promoting appropriate immune responses to pathogens and maintaining proper immune homeostasis (Maynard et al., 2012). Germ-free mice, which lack a microbiota, have poorly developed innate and adaptive immune systems. Germ-free mice do not have a mucous layer, and AMP production, sIgA secretion, and barrier-protective IL-22/IL-17A expression are downregulated, limited in function, or undetectable (Round and Mazmanian, 2009). Similar to CD4^+^ T cell numbers, intestinal CD8^+^ T cell numbers are markedly reduced in germ-free or antibiotic-treated mice compared to SPF animals (Tanoue et al., 2019). Accordingly, colonization with a defined consortium of human commensals restores the deficit in colonic IFN-γ-producing CD8^+^ T cells in germ-free mice. Furthermore, SPF laboratory mice lack effector-differentiated and mucosally distributed memory T cells, however, wild pet store mice have these memory cell populations and had a much more diverse microbial experience (Beura et al., 2016). Thus, wild or pet store mice reflect the adult human situation more accurately than SPF mice. It has been reported that bifidobacteria can promote vaccine efficacy by enhancing immunological memory. Infants with a relatively high abundance of bifidobacteria have relatively robust CD4^+^ T-cell responses against BCG and tetanus toxoid at 15 weeks and 2 years of age (Huda et al., 2019).

Recently, several studies have demonstrated that the gut microbiome can improve the therapeutic efficacy of chemotherapeutic drugs and blocking ICI antibodies, and potentially limit ICI immune-mediated toxicity (Gopalakrishnan et al., 2018a; Gopalakrishnan et al., 2018b; Routy et al., 2018; Riquelme et al., 2019), furthermore, the gut microbiome can induce memory T cell formation during the process of the tumor elimination (Viaud et al., 2013). It has been reported that the gut microbiota contributes to the antitumor effect of cyclophosphamide partly by stimulating memory T cell formation. It has been found that cyclophosphamide can change the composition of gut microbes, reducing of lactobacilli and enterococci levels, and inducing gram-positive bacterial transfer to the secondary lymphoid organs, and that the microbes in the secondary lymphoid organs can stimulate the host to produce a group of special ‘pathogenic’ Th17 cells (pTh17) and an immune reaction involving memory Th1 cells (Viaud et al., 2013). There are memory CD4^+^ T cells specific to some Bacteroides species in the spleens of anti-CTLA-4 mAb treated mice and in the blood of ipilimumab-treated cancer patients (Pitt et al., 2016). Data from 37 advanced NSCLC patients receiving nivolumab enrolled in clinical trials have demonstrated a strong correlation between the level of gut microbiome diversity and anti-PD-1 antibody efficacy in advanced NSCLC Chinese patients (Jin et al., 2019). Patients with high gut microbiome diversity (reported as favorable gut microbiome) exhibited increase in memory T and NK cell signatures in peripheral blood samples. Routy et al. found that antibiotics compromise the efficacy of PD-1 blockade in mouse tumor models and cancer patients. Reconstitution with commensals such as *A*. *muciniphila* and *E*. *hirae* reversed resistance to PD-1 blockade in antibiotic-treated mice. Reconstitution with immune-sensitizing microbes was associated with the accumulation of central memory CCR9^+^CXCR3^+^ CD4^+^ T cells in an IL-12-dependent manner, resulting in an incremental increase in CD4/Foxp3 ratios (Routy et al., 2018). An outstanding study conducted by the Kenya Honda group reported that a consortium of 11 bacterial strains from healthy human donor feces could potentially induce IFN-γ-producing CD8^+^ T cells and enhance the therapeutic efficacy of immune checkpoint inhibitors (anti-PD-1 and anti-CTLA4 antibodies) in syngeneic tumor models (MC-38 tumor and melanoma) in a manner, dependent on CD103^+^ DCs and MHC-I molecules. Furthermore, memory T cell phenotypes were observed (Tanoue et al., 2019). Vétizou found that memory T cells specific to *B*. *thetaiotaomicron* or *B*. *fragilis* were associated with the efficacy of an anti-CTLA-4 antibody (Vetizou et al., 2015). These findings provide important implications for the role of gut microbiome diversity in memory T cell (especially memory CD4^+^ T cells) formation in chemotherapy and ICI immunotherapy responses.

An attenuated *Salmonella enterica* serovar typhimurium strain can deliver heterologous antigens to induce cytotoxic effector and memory CD8^+^ T cell responses resulting in efficient prevention of tumor growth. Furthermore, it has been demonstrated that immunization elicits high frequencies of peptide-specific CD8^+^ Tcm and Tem cells in the spleen, whereas in the blood the majority of peptide-specific lymphocytes belonged to the Tem and Teff CD8^+^ cell subsets (Panthel et al., 2006). Thus, the bacterial type III secretion system can be used for heterologous antigen delivery to induce cytotoxic effector and memory CD8^+^ T cell responses, resulting in efficient prevention of tumor growth.

The mechanisms underlying memory T cell formation induced by the gut bacteria may partly involve the mucosal immune system to sensing the microbiota through several receptors resulting in cytokine or chemokine production. For example, NLRP6 mediates the activation of mucus secretion (Wlodarska et al., 2014), AHR mediates IL-22 production (Monteleone et al., 2011), MyD88 mediates microbiota-specific IgA secretion (Kubinak et al., 2015), TLRs mediate AMP secretion(Zhang and Gallo, 2016), and GPCRs mediate T cell activation (Wang, 2018). Colonizing microbes phagocytosed by DCs stimulate DCs to release proinflammatory cytokines such as IL-6, TNF, OX40 and IL-12. Noninvasive microbiota species can provide multiple signals that influence the generation of Tregs, which is mediated by goblet cells and CD103^+^ DCs combined with high levels of TGF-β and IL-10. Furthermore, receptor activation is tightly regulated and dependent on the cell type, the amplitude of the signal, and the location in the tissue and microenvironment (Ignacio et al., 2016). Thus, the presence, absence, or amplitude of microbiota-induced factors drives polarization of antigen-specific memory T cells (Brown et al., 2019). Several studies demonstrated that gut microbiota catabolism regulates the differentiation of memory T cells. SCFAs and tryptophan metabolites are synthesized by clostridium and sensed by GPCRs and AHRs, respectively, on both T cells and DCs, which also directly promotes Tregs differentiation. It has been reported that memory CD8^+^ T cells are susceptible to tryptophan catabolism mediated by IDO. Overexpression of IDO *in vivo* can attenuate the generation and function of both Tcm CD8^+^ cells and Tem CD8^+^ cells, while suppressing IDO activity promoted their generation of these subsets (Liu et al., 2007). Intracellular L-arginine concentrations directly impact the metabolic fitness and survival capacity of T cells, and promote the generation of central memory-like cells endowed with a relatively high survival capacity and, in a mouse model, antitumor activity. Moreover, arginine supplementation during the *in vitro* expansion of T cells promotes T cell differentiation into Tcm-like cells with superior antitumor activity (Geiger et al., 2016). A study demonstrated that the microbiota-derived short-chain fatty acid (SCFA), butyrate, promoted cellular metabolism, and enhanced the memory potential of activated CD8^+^ T cells. SCFAs were required for optimal recall responses upon antigen re-encounter. Mechanistic experiments revealed that butyrate uncoupled the tricarboxylic acid cycle from glycolytic input in CD8^+^ T cells, which allowed preferential fueling of oxidative phosphorylation through sustained glutamine utilization and fatty acid catabolism (Bachem et al., 2019). ILC3s express CD30L and OX40L to prevent inflammatory responses while also supporting memory cell responses necessary for optimal immunity through regulating host-commensal bacteria interactions (Goc et al., 2015). There are commensal-specific memory T cells in the peripheral blood and mucosal samples of healthy people, which are capable of expressing different cytokines according to their immune specificities (Hegazy et al., 2017; Sorini et al., 2018). Furthermore, the intestinal microbiota also modulates IgA production through the generation of ATP which mediates P2X7 signaling in T follicular helper cells (Proietti et al., 2014; Proietti et al., 2019). Thus, we speculate that enhancement of the efficacy of antitumor therapy may be due to the activation of memory T cells by crosstalk between gut microbes and various immune cells. This suggests that targeting gut bacteria in conjunction with immunotherapy could boost the success rates of drugs.

In summary, the gut microbiota may enhance the functions of DCs by increasing the potency of tumor antigen presentation and cytokine production, increase the formation of memory T cells and their trafficking from the mesenteric and draining lymph nodes to the TME, decrease Treg and MDSC numbers, and increase the recruitment and activation of IFN-γ-producing tumor antigen-specific effector T cells which together contribute to the modulation of the antitumor immune response.

## Manipulation to produce memory T cells *in* (*ex*) *vivo*

Because memory T cells play important roles in antitumor therapy, several strategies might be developed to promote the phenotypes and functions of memory T cells *in* (*ex*) *vivo* based on the mechanisms underlying natural memory T cell formation (Figs. 2 and 3).

It is well accepted that IL-7 and IL-15 are involved in maintaining the numbers of CD8^+^ memory cells *in vivo* (Klonowski and Lefrancois, 2005; Surh and Sprent, 2005; Ma et al., 2006; Cieri et al., 2013). An *in vitro* system based on IL-7 and IL-15 that leads to the generation of Tcm-like CD8^+^ T cells has been developed (Carrio et al., 2004). We also found that IL-15 promoted the generation of human Tcm-like CD4^+^ T cells more easily than Tcm-like CD8^+^ T cells. IL-7 has been shown to be as efficient as IL-15 in promoting the differentiation of cells with memory phenotypes. Naïve T cells as well as memory CD8^+^ T cells express the IL-7 receptor, and stimulation through this receptor is thought to result in prosurvival signaling in both subsets. The contributions of IL-15 to memory are more specific, and more complex than those of IL-7. It has been reported that IL-27 and IL-15 are critical for subunit vaccine-elicited T cell responses and function through a mitochondrial metabolic program (Klarquist et al., 2018). Recently, Adachi et al. engineered IL-7 and CCL19-expressing CAR-T cells, which mediated memory responses against tumors and resulted in complete regression of pre-established solid tumors and prolonged mouse survival (Adachi et al., 2018). A specific memory T cell culture system developed by Butler et al. included artificial antigen-presenting cells (aAPCs), IL-2 and IL-15, which could generate specific antitumor Tcm and Tem cells with the capacity to survive for prolonged periods of time *ex vivo* (Butler et al., 2007; Gomez-Eerland et al., 2014). They showed that these CTLs trafficked to tumors, mediated biological and clinical responses, and established antitumor immunological memory (Butler et al., 2011).

The mTOR-Akt pathway plays an important role in the formation of memory T cells. Accordingly, mTOR inhibitors (mTORi) permit the differentiation of naïve T cells into cells with a memory phenotype and allow the production of IL-2 (Setoguchi et al., 2015; Merino et al., 2016). However, IL-2 production driven by mTORi also allows the differentiation of naïve T cells into Treg cells (Battaglia et al., 2005; Kopf et al., 2007; Wang et al., 2012). Rapamycin has been demonstrated to have an immunostimulatory effect on generation of memory CD8^+^ T cells (Araki et al., 2009). The requirement for mTOR-Akt pathway signaling was confirmed by Merino et al. using tacrolimus, which permitted sorted naïve T cells to differentiate into Tcm cells, and rapamycin, which produced not only Tcm phenotypes but also Tem phenotypes (Merino et al., 2016). They also found that the effects of rapamycin on phenotypic changes in Tcm and Tem cells were observed even at low concentrations. In contrast, rapamycin was able to control the proliferation of memory T cells only at a high concentration (Merino et al., 2016). The mTOR pathway is well-documented to promote continuous production of IFN-γ by memory T cells (Setoguchi et al., 2015). However, rapamycin fails to control IFN-γ production by memory T cells (Merino et al., 2016). Agonist TIL Akt inhibition enhances the expansion of potent tumor-specific lymphocytes with memory cell characteristics (Crompton et al., 2015). It is important to note that a nonapoptotic Fas signal, which results in Akt-driven cellular differentiation, mediates the conversion of naïve T cells into memory T cells. Thus, Fas signaling blockade preserves the antitumor activity of naïve cells within mixed cell populations (Dolfi et al., 2008; Klebanoff et al., 2016). A high potassium (K^+^) concentration impairs TCR-driven Akt-mTOR phosphorylation and the effector program. Accordingly, lowering the K^+^ concentration improves effector function *in vitro* and *in vivo*, and enhances tumor clearance and survival in melanoma-bearing mice (Eil et al., 2016). Thus, controlling Akt/mTOR signaling may represent a new strategy for forming memory T cells.

KLRG1 is induced in highly cytotoxic and proliferative effector CD8^+^ T cells that receive strong cumulative TCR and inflammatory signals. It has been demonstrated that KLRG1^+^IL-7Ra^+^ effector CD8^+^ T cells downregulate KLRG1 expression in a Bach2-dependent manner and differentiated into long-lived circulating and tissue-resident ‘‘exKLRG1’’ memory cells. These memory T cells retain high cytotoxic and proliferative capacities distinct from those of other populations, and contributed to effective anti-influenza and anti-tumor immunity (Herndler-Brandstetter et al., 2018). Furthermore, Hu et al. showed that a synthetic, small-molecule RORγ agonist could potentiate the antitumor activities of human Th17 and Tc17 cells since RORγ agonists conferred durable memory and stemness *in vivo* (Hu et al., 2018). Thus, regulating KLRG1 or RORγ may be one method to induce a memory T cells phenotype. It was recently reported that P2RX7 is required for the establishment, maintenance and functionality of long-lived central and tissue-resident memory CD8^+^ T cell populations (Borges da Silva et al., 2018; Greene et al., 2018). P2RX7 is a purinergic receptor and is used for sensing extracellular adenosine triphosphate. According to the study conclusion, we speculate that ATP will be a good reagent to induce a memory T cell phenotype.

The gut microbiota and associated catabolites promote memory T cells formation and enhance the antitumor efficacy of blocking ICI antibodies. Thus, improvement in the constitution of the gut microbiota of tumor patients by some approaches or replenishment of some catabolites such as arginine *ex vivo*, could represent attractive antitumor strategies. Vancomycin treatment induces an increase in systemic CD8α^+^ DC numbers, which sustains adoptively transferred antitumor T cells systemically in an IL-12-dependent manner. It has been demonstrated that the gut microbiota plays an important role in the antitumor effectiveness of ACT (Uribe-Herranz et al., 2018). Therefore, altering the gut microbiota is a promising way to improve the response to ACT therapy. In mouse tumor models studies, the arginine inhibitor INCB001158 increased CD8^+^ T cell and NK cell tumor infiltration and stimulated the production of inflammatory cytokines in the TME (Steggerda et al., 2017). INCB001158 in combination with the immune checkpoint inhibitor antibody pembrolizumab is currently being evaluated in a clinical trial (Blankenstein et al., 2012). Thus, tryptophan and L-arginine could also be used for inducing memory T cell phenotypes.

Overall, we can manipulate T cells to induce memory T cell phenotypes in many ways such as cytokines administration, Akt/mTOR regulators administration, KLRG1, RORγ or P2RX7 regulation, microbiome manipulation and catabolism regulation.

## Concluding remarks

Immunotherapy is a promising treatment approach for advanced cancers. ACT and blocking ICI antibodies (Anti-PD-1 and anti-CLTL-A4 antibodies) represent two major potentially curative treatments for patients with advanced cancer. The progress in the ability to genetically redirect patient-derived peripheral blood T cells toward tumors by modification with antigen specific TCRs or CARs has greatly simplified the generation of therapeutic T cells. Given the clinical efficacy of T cell therapy combined with the ability of T cells to be manufactured according to standardized procedures, ACT is now poised to enter mainstream clinical practice. However, the majority of clinical experiences with ACT have used cells extensively expanded *ex vivo*, generating cells with Tem or Teff phenotypes for cell transfer (Powell et al., 2005; Pule et al., 2008; Johnson et al., 2009). These effector phenotypes have been a suboptimal predictor of *in vivo* performance, potentially because the *ex vivo* expansion process drives cells to take on an overmanipulated, exhausted phenotype (Dudley et al., 2002; Yee et al., 2002; Klebanoff et al., 2006).

ACT of purified naïve Tscm, and Tcm cell subsets results in superior persistence and antitumor immunity compared with ACT of populations containing Tem and Teff cells (Golubovskaya and Wu, 2016). Thus, other immunotherapy strategies resulting in memory T cell formation could produce better antitumor efficacy than current strategies (Fig. 2). This review systematically discussed the efficacy against tumors, cell origin, formation mechanisms of memory T cells, and the role of the gut microbiota in memory T cell formation. We hope to reveal the molecular pathways driving memory T cell formation, which will be essential in the development of rational approaches to optimize cancer immunotherapy. This work will greatly help us to optimize the source, expansion, and quality of therapeutic T cells used for transfer. Better exploitation of homeostatic proliferation, during conditioning regimens to expand memory-phenotype T cells *ex vivo* as described above, may provide additional options for human ACT protocols (Fig. 3). Furthermore, blocking ICI antibodies can block the inhibitory effect of tumor cells on T cells, and some gut microbes play important roles in promoting and maintaining memory T cells. Thus, blocking ICI antibodies and gut microbiota-based reagents will broaden the availability of ACT for patients, both alone and in combination with other therapeutic modalities.
